# Deep learning MRI halves scan time and preserves image quality across routine neuroradiologic examinations

**DOI:** 10.1093/radadv/umaf029

**Published:** 2025-08-23

**Authors:** Shawn K Lyo, Suyash Mohan, Michael J Hoch, Vivek P Patel, Robert M Kurtz, Alvand Hassankhani

**Affiliations:** Department of Radiology, Hospital of the University of Pennsylvania, Philadelphia, PA 19104, United States; Department of Radiology, Hospital of the University of Pennsylvania, Philadelphia, PA 19104, United States; Department of Radiology, Thomas Jefferson University Hospital, Philadelphia, PA 19107, United States; Department of Radiology, Hospital of the University of Pennsylvania, Philadelphia, PA 19104, United States; Department of Radiology, Hospital of the University of Pennsylvania, Philadelphia, PA 19104, United States; Department of Radiology, Hospital of the University of Pennsylvania, Philadelphia, PA 19104, United States

**Keywords:** deep learning MRI reconstruction, deep learning MRI, MRI reconstruction, MRI, neuroradiology, scan time reduction, image quality, hybrid imaging protocols

## Abstract

**Background:**

Magnetic resonance imaging (MRI) is a cornerstone of neuroimaging but is limited by lengthy acquisition times, which can lead to motion artifacts, patient discomfort, and delayed care. Deep learning reconstruction is an emerging technology that can offer image acquisition acceleration while maintaining image quality.

**Purpose:**

To compare image quality and acquisition efficiency between deep learning–accelerated vs conventional MRI (C-MRI) across a spectrum of routine neuroradiologic examinations.

**Materials and Methods:**

In this single-center retrospective study, 26 patients underwent imaging with a commercially available, FDA-cleared deep learning-accelerated MRI reconstruction algorithm (Deep Resolve, Siemens Healthineers), and C-MRI on a Siemens 3 T MAGNETOM Vida scanner between October 24 and November 14, 2023. A total of 113 sequence pairs were acquired across multiple body parts (brain [*n* = 28], cervical spine [*n* = 24], thoracic spine [*n *= 16], lumbar spine [*n* = 14], internal auditory canals [*n* = 5], sella [*n* = 5], neck [*n* = 5], temporomandibular joints [*n* = 6], brachial plexus [*n* = 4], and orbits [*n* = 6]) and sequences (T2 [*n* = 38], T1 [*n* = 30], short tau inversion recovery [*n* = 21], T1 post-contrast [*n* = 17], T2 fluid attenuated inversion recovery [*n* = 5], and proton density [*n *= 2]) and evaluated by 4 neuroradiologists blinded to the acquisition method for image quality using a 5-point Likert scale. Acquisition parameters were extracted from Digital Imaging and Communications in Medicine (DICOM) metadata and statistically compared. Rater preferences and interrater reliability were assessed using nonparametric tests and intraclass correlation coefficients.

**Results:**

Deep learning reduced mean scan time by 51.6% (95% CI: 45.7%–57.7%; from 110.8 seconds to 53.7 seconds; *P* < .001). Image quality assessments using a Likert scale showed scores slightly above neutral for signal-to-noise ratio (mean 3.51; 95% CI: 3.44–3.58), structural delineation (mean 3.51, 95% CI: 3.44–3.56), and overall image quality (mean 3.56, 95% CI: 3.49–3.63). However, poor interrater reliability (intraclass correlation [ICC] range: 0.06–0.33) showed that the observed differences were not consistent, indicating functional equivalence between conventional and deep learning images.

**Conclusion:**

Deep learning MRI enabled substantial scan time reductions while maintaining image quality.

**Abbreviations**

C-MRI = conventional MRI; DICOM = Digital Imaging and Communications in Medicine; DL = deep learning; DL-MRI = deep learning-derived MRI; HIPAA = Health Insurance Portability and Accountability Act; ICC = intraclass correlation; MRI = magnetic resonance imaging; SNR = signal-to-noise ratio; STIR = short tau inversion recovery; TSE = turbo spin echo

**Summary**

Deep learning MRI reduced scan times by 51.6% on average across routine neuroradiologic examinations while maintaining image quality.

**Key Results**

- While time savings varied by sequence, deep learning MRI reduced scan time by 51.6% (95% CI: 45.7%–57.7%) compared to conventional MRI across routine neuroradiologic examinations.
- Overall image quality was maintained, with no reader preference seen between conventional and accelerated examinations.
- Distinct artifact patterns were observed with deep learning MRI compared to conventional MRI.

## Introduction

Magnetic resonance imaging (MRI) is a cornerstone of medical imaging that affords remarkable tissue contrast resolution without the risks of ionizing radiation and is the diagnostic imaging modality of choice for various pathologies. However, its utility is hampered by lengthy scan times,^1^^,^^2^ which can pose significant challenges to numerous patient demographics, including pediatric patients,^3^ critically ill or claustrophobic patients,^4^ and patients with MRI conditional devices/implants.^5^ The need for patients to remain still for extended periods also increases the risk of motion artifacts and may necessitate sedation in select cases. Furthermore, faster imaging allows for increased patient throughput.

Methods of accelerating MRI scans often risk trade-offs, sacrificing either image resolution or signal-to-noise ratio (SNR), thereby affecting the diagnostic quality of the images. Techniques such as parallel imaging^6^^,^^7^ and methods of k-space undersampling, such as compressed sensing (CS),^8–10^ have marked important milestones, enabling faster acquisition by leveraging the spatial distribution of receive coil arrays and the sparsity of MR images in the transform domain, respectively.

Deep learning (DL) is a subdomain of artificial intelligence that has been used to reconstruct k-space undersampled images.^11–14^ Deep learning allows greater parallel acceleration ratios and higher-quality image reconstructions than non-deep-learning approaches.^11^^,^^15^^,^^16^ Numerous implementations of deep learning MRI image optimization exist, including image postprocessing packages functioning on the image domain, such as SubtleMR (Subtle Medical, Inc., Menlo Park, CA, USA), or image reconstruction packages offered by MRI vendors which process k-space and/or related transform domains, such as Deep Resolve (Siemens Healthineers, Erlangen, Germany) and AIR Recon DL (GE Healthcare, Milwaukee, WI, USA), among others.

Deep learning-derived MRI (DL-MRI) has shown effective scan time reduction with satisfactory image quality in neuroradiologic examinations.^17–20^ Bash et al.^21^ and Kim et al.^22^ showed that deep learning can enhance image quality and reduce scan times by up to 60% for adult brain and pediatric neuroimaging without compromising diagnostic integrity. Similarly, advancements in spinal MRI and imaging of the orbit have reduced scan time by 40% and 69%, respectively, with simultaneous improvements in image quality and reduction of artifacts.^23–26^ These studies demonstrated the efficacy of DL-MRI on specific examinations. However, there is limited data on the broad application of DL-MRI across a spectrum of neuroimaging examinations in a clinical setting and which sequences and exam types are most suited for DL-MRI.

We conducted a head-to-head comparison between DL-MRI and conventional MRI (C-MRI), assessing the feasibility of DL-MRI across a spectrum of neuroradiologic studies in a busy tertiary care academic medical center. Our primary aim was to systematically evaluate whether DL-MRI provides clinically meaningful reductions in scan time without compromising diagnostic image quality within the practical constraints and heterogeneous patient population of a busy academic practice. A secondary, exploratory aim was to preliminarily identify and characterize sequences or anatomical regions that might benefit disproportionately from DL-MRI, to help inform future studies and protocol optimization.

## Materials and methods

### Study design and participants

This retrospective single-center cross-sectional study compared MRI sequences obtained from 26 patients who underwent DL-MRI and C-MRI on a 3 T MAGNETOM Vida Siemens Healthineers scanner between October 24, 2023 and November 14, 2023, initially performed for internal quality improvement. During this quality improvement initiative, patients were selected based on scanner time availability to accommodate both conventional and deep learning acquisitions within a single examination session. This scheduling-based selection yielded 26 patients with 113 paired sequences. All acquired paired sequences were included in the analysis without exclusion. The study encompassed multiple 2D turbo spin echo (TSE) sequences from a sample of routine neuroimaging examinations, including the brain, internal auditory canals, pituitary/sella, orbits, neck, temporomandibular joints, brachial plexus, and cervical, thoracic, and lumbar spines. Post-hoc power analysis indicated that for (1-ß) = 0.9 and α  =  0.05, our sample of 113 pairs would be sufficient to demonstrate a moderate effect (Cohen d ≥ 0.31 for continuous variables; r ≥ 0.30 for non-parametric tests). Institutional Review Board (IRB) approval was obtained with a waiver of informed consent for this retrospective study. All imaging data were de-identified in accordance with Health Insurance Portability and Accountability Act (HIPAA) regulations. None of the patients included in this cohort has been previously reported.

### MRI acquisition and parameter comparison

The DL-MRI sequences were acquired using Deep Resolve (Siemens Healthineers, Erlangen, Germany), a commercially available, FDA-cleared algorithm, which applies wavelet space denoising and resolution enhancement via deep learning networks.^27^ This allows for greater k-space undersampling while maintaining image quality through learned reconstruction. C-MRI sequences were acquired using standard, fully-sampled acquisitions. The DL-MRI reconstruction algorithm utilized a proprietary convolutional neural network with fixed parameters. User modifications to the DL package were limited to parallel acceleration factor and number of averages. We performed minor optimization of standard parameters (eg, repetition time [TR], echo time [TE], saturation band placement, etc.) based on preliminary image review. All sequences were TSE acquisitions that differed only in their sampling and reconstruction approaches. The order of acquisition was not completely standardized; however, C-MRI and DL-MRI sequences were predominantly acquired in an interleaved fashion (eg, C-MRI sagittal T2 followed by DL-MRI sagittal T2). In some instances, the complete set of C-MRI sequences was acquired prior to DL-MRI sequences. Acquisition parameters were extracted from Digital Imaging and Communications in Medicine (DICOM) headers to compare C-MRI and DL-MRI sequences and assess changes potentially affecting image quality. The extracted imaging parameters for all sequence pairs are provided in Supplementary Data File S1.

### Image pairing and rating

Three attending neuroradiologists (4, 8, and 23 years of experience) and 1 neuroradiology fellow evaluated each of the 113 pairs of sequences acquired with C-MRI and DL-MRI while being blinded to the acquisition method. At the time of this study, DL-MRI was in early clinical implementation at our institution and all raters had at least some prior exposure, with varying levels of familiarity. Whole sequences were anonymized and displayed side by side using fully interactive web DICOM viewers (Orthanc^28^). The order of presented sequences and the presence of C-MRI or DL-MRI on either the left or right were dynamically randomized to minimize bias using a pseudorandom number generator without a fixed seed. Sequence order was randomized using a uniform distribution without patient-specific grouping. Image quality was rated on a 5-point Likert scale, where 3 indicated no preference between sequences, 1 indicated a strong preference for the sequence on the left, and 5 indicated a strong preference for the right (Figure S1). The raters were provided written instructions and indicated preferences between the 2 sequences on multiple parameters, including perceived signal-to-noise ratio, diagnostic quality, artifacts, overall preference, structural delineation, and characterization of various tissues including fat, muscle, marrow, and neural structures (Table S1). No formal calibration was performed as the assessments were based on the degree of preference. Raters were also able to provide freeform text comments regarding the sequence pairs.

### Statistical analysis

Statistical analysis was performed using Python and data analysis libraries. The analysis included multiple univariate and multivariate tests, including paired t-tests for comparing acquisition parameters, a Wilcoxon signed rank test to evaluate sequence rating preferences against a median expectation, and intraclass correlation coefficients to assess interrater reliability. The effect size (r) of the Wilcoxon signed rank test was calculated as $r=\frac{z}{\sqrt{n}}$, where z is the standardized test statistic and n is the total number of observations. Effect sizes were interpreted as follows: 0.1 – <0.3 (small effect), 0.3 – <0.5 (moderate effect) and ≥ 0.5 (large effect). Interrater reliability was computed utilizing a single fixed raters model intraclass correlation (ICC). A *P*-value of .05 was set as the threshold for statistical significance. Bonferroni correction was applied as appropriate for strict rejection of type I error.

## Results

### Patient demographics and acquired sequence characteristics

A total of 113 pairs of sequences were acquired from 26 patients (mean age: 54.5 years, SD = 14.3; 17 women [65.4%], Table 1) using both C-MRI and DL-MRI across various neuroradiologic examinations (Table 2, Table S2). The most frequently imaged anatomical region was the brain (28 pairs), followed by the cervical (24 pairs), thoracic (16 pairs), and lumbar spine (14 pairs). Most of the sequences were acquired in either the axial (47 pairs) or sagittal plane (48 pairs). Most sequence pairs were T2-weighted (64), including T2 Fluid-Attenuated Inversion Recovery (FLAIR) and short Tau inversion recovery (STIR). Forty-seven pairs were T1-weighted, with 14 acquired post-contrast (Table S2).

**Table 1.** Patient demographics.

Characteristic	Value
Patients, *n*	26
Age, mean (SD), years	54.5 (14.3)
Sex, *n* (%)	
Female	17 (65.4)
Male	9 (34.6)

**Table 2.** Study characteristics.

Characteristic	Value
Body parts, *n* (%)	
Brain	28 (24.8)
Cervical spine	24 (21.2)
Thoracic spine	16 (14.2)
Lumbar spine	14 (12.4)
Orbits	6 (5.3)
TMJ	6 (5.3)
IAC	5 (4.4)
Sella	5 (4.4)
Neck	5 (4.4)
Brachial plexus	4 (3.6)
Orientations, *n* (%)	
Axial	48 (42.5)
Sagittal	47 (41.6)
Coronal	18 (15.9)
Sequences, *n* (%)	
T2	39 (34.5)
T1	30 (26.5)
STIR	20 (17.7)
T1 post contrast	17 (15.0)
T2 FLAIR	5 (4.4)
PD	2 (1.8)

Abbreviations: FLAIR, fluid-attenuated inversion recovery; IAC, internal auditory canals; PD, proton density; STIR, short Tau inversion recovery; TMJ, temporomandibular joint.

### Scanning parameters

**Figure 1.**
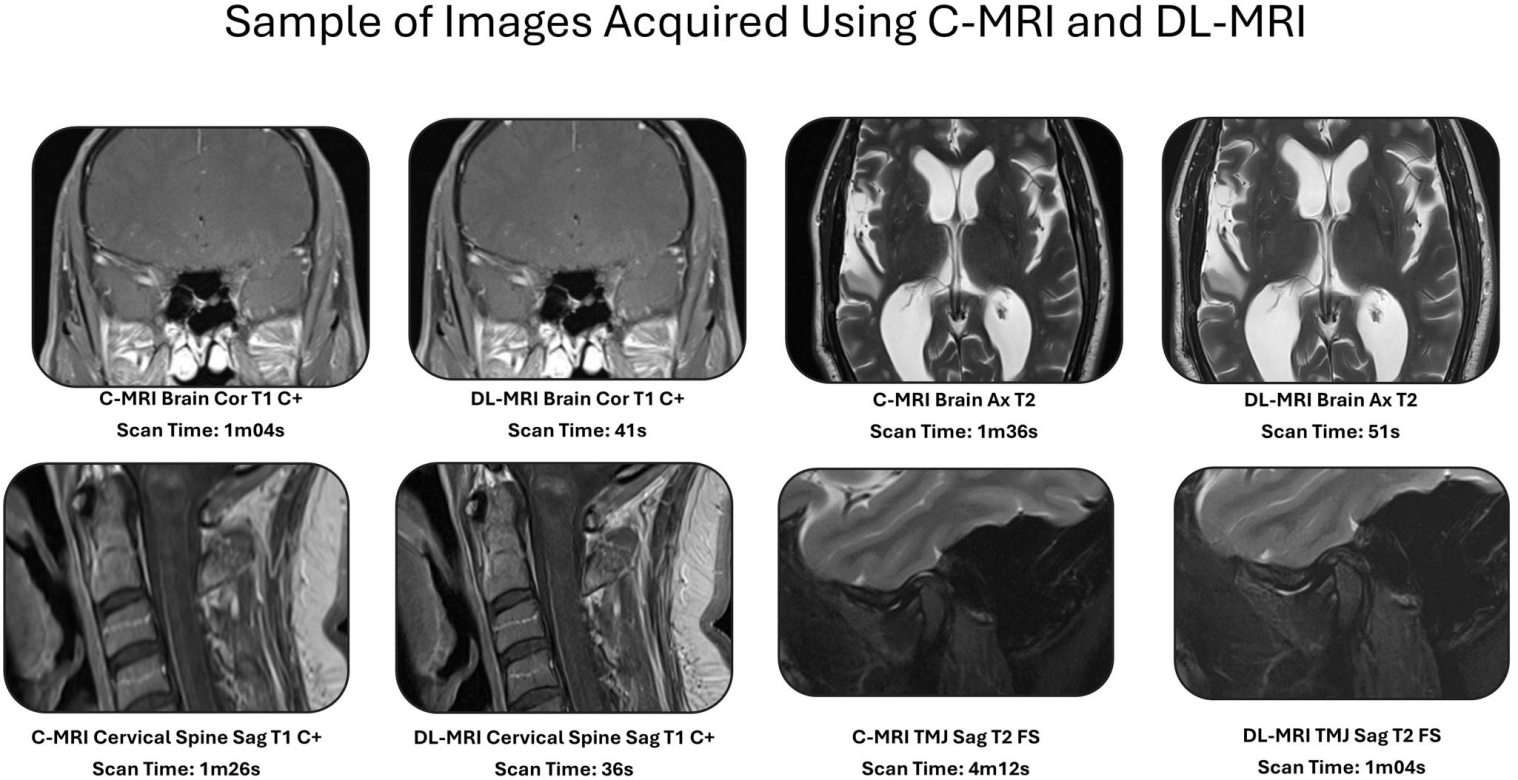
Sample paired images acquired using conventional MRI (C-MRI) and deep learning-based MRI (DL-MRI) with respective scan times. Abbreviations: Ax, axial; C+, contrast enhanced; C-MRI, conventional MRI; Cor, coronal; DL-MRI, deep learning MRI; FS, fat suppressed; Sag, sagittal.

**Figure 2.**
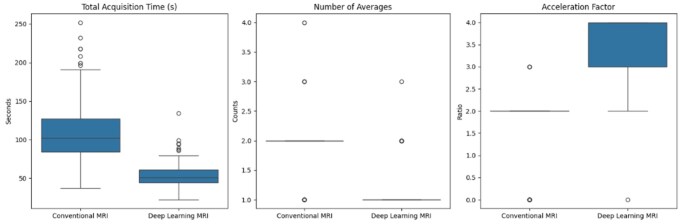
Boxplots comparing scan parameters in DL-MRI and C-MRI. DL-MRI scan time was markedly reduced compared to C-MRI, related to increased acceleration factor and fewer averages. Abbreviations: C-MRI, conventional MRI; DL-MRI, deep learning MRI.

Representative examples of image quality and scan time reduction are shown in Figure 1. DL-MRI sequences were acquired approximately 51.6% (95% CI: 45.7%–55.7%) faster than C-MRI, requiring an average of 53.65 seconds (SD 17.45) compared to 110.84 seconds (SD 40.64) for C-MRI (t(113) = −16.8, *P* < .001; Table S3; Figure 2). The increased speed was due to significantly increased mean parallel acceleration factor of 3.58 (SD 0.65) in DL-MRI compared to 1.54 (SD 0.90) in C-MRI (*P* < .001; Figure 2), as well as a significantly lower mean number of averages (1.11 (SD 0.34) vs 1.88 (SD 0.53); *P* < .001; Figure 2). No other parameters reached statistical significance following Bonferroni correction (Table S3). The observed effect size for scan time reduction was large (Cohen d = 1.58) and exceeded the minimum detectable effect size of 0.31 identified in our post-hoc power analysis.

### Acquisition time by study characteristics

DL-MRI sequences demonstrated a marked decrease in acquisition time, with the most significant reduction in acquisition time in sequences evaluating the sella (Figure S2). Sequences acquired in the coronal plane also demonstrated greater scan time reduction than sagittal and axial sequences (Figure S2).

### Rater preference

**Figure 3.**
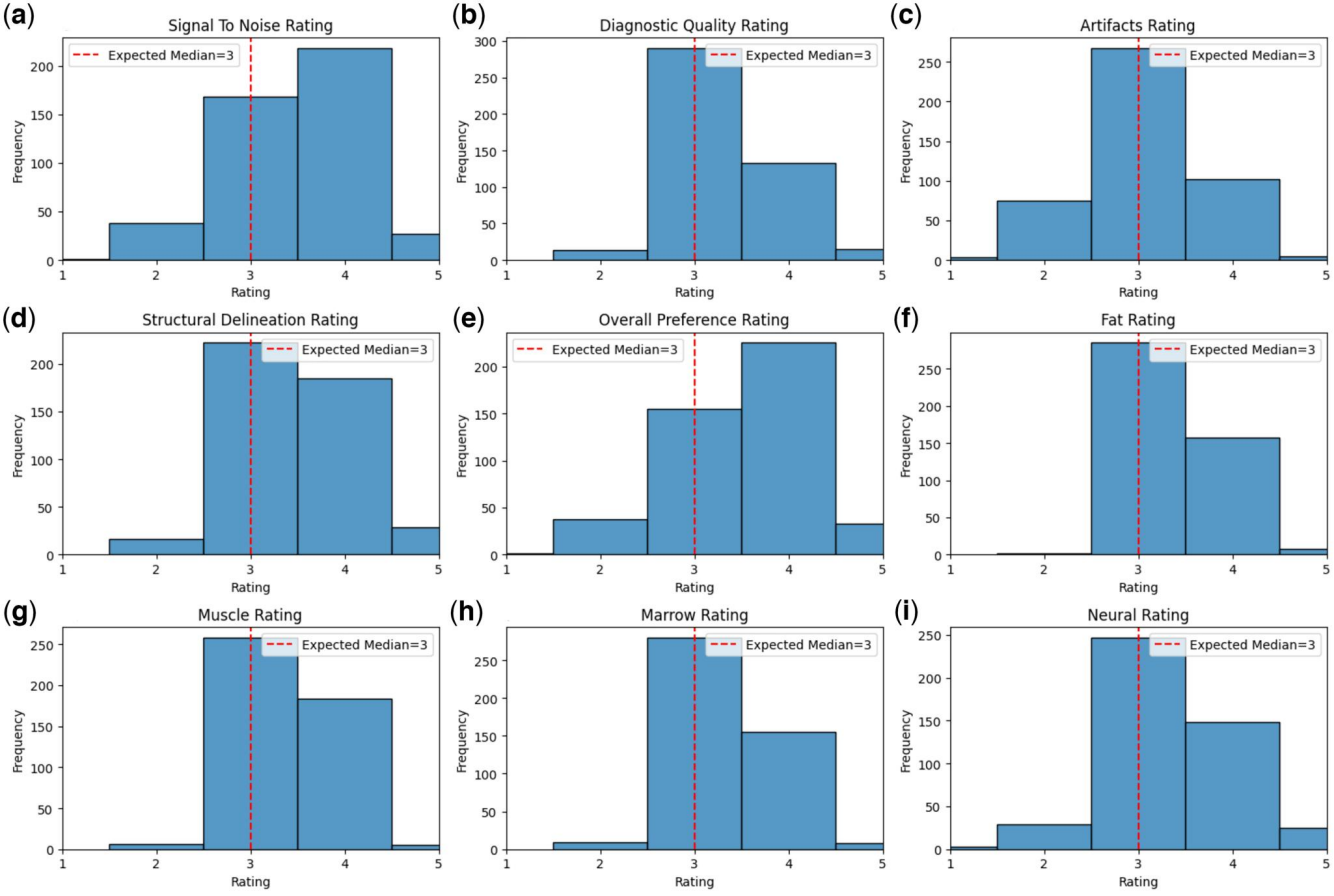
Histograms demonstrating the frequency of adjusted rater responses when comparing DL-MRI to C-MRI sequences on multiple parameters, including perceived signal-to-noise rating (A), diagnostic quality (B), presence of artifacts (C), structural delineation (D), overall preference (E), and characterization of various tissues including fat (F), muscle (G), marrow (H), and neural structures (I). Higher numbers indicate a preference for DL-MRI, while lower numbers indicate a preference for C-MRI. There was a significant preference for DL-MRI in all parameters except for artifacts. Abbreviations: C-MRI, conventional MRI; DL-MRI, deep learning MRI.

Multiple Wilcoxon rank sum tests demonstrated a modest preference for DL-MRI over C-MRI on all assessed parameters except for artifacts (Table 3, Figure 3). The mean rating for overall preference was 3.56 (95% CI: 3.49–3.63); 3.51 for signal-to-noise ratio (95% CI: 3.44–3.58), 3.50 for structural delineation (95% CI: 3.44–3.56), and 3.33 for diagnostic quality (95% CI: 3.27–3.38). There was no significant preference for artifacts with a mean rating of 3.06 (95% CI: 3.00–3.13). The effect size was large for all significant parameters (*r* = 0.61–0.85; Table 3) except for artifacts (*r* = 0.14). The statistically significant effect sizes exceeded the minimum detectable threshold of 0.30 from our post-hoc power analysis.

**Table 3.** Analysis of image quality ratings comparing DL-MRI to C-MRI.

Adjusted rating category	Average preference (95% CI)	Wilcoxon rank sum	Effect size ($\frac{z}{\sqrt{n}}$)	*P*-value
Signal-to-noise	3.51 (3.44–3.58)	5153.5	0.65	<.001
Diagnostic quality	3.33 (3.27–3.38)	1036.0	0.73	<.001
Artifacts	3.07 (3.00–3.13)	7219.5	0.14	.03
Structural delineation	3.50 (3.44–3.56)	1616.0	0.76	<.001
Overall preference	3.56 (3.49–3.63)	5296.5	0.66	<.001
Fat	3.38 (3.31–3.45)	160.0	0.85	<.001
Muscle	3.41 (3.34–3.48)	570.0	0.81	<.001
Marrow	3.36 (3.29–3.43)	742.5	0.78	<.001
Neural	3.36 (3.29–3.43)	3155.5	0.61	<.001

Raters indicated their preference between paired sequences for: perceived signal-to-noise ratio, perceived diagnostic quality of the images, artifacts (less degradation = better), delineation of anatomical structures, overall global preference, and characterization of different types of tissues (fat, muscle, marrow, and neural structures). For average preference, 3 indicates equivalence between DL-MRI and C-MRI, numbers greater than 3 indicate preference for DL-MRI, and numbers less than 3 indicate preference for C-MRI. Following Bonferroni correction for multiple comparisons, adjusted criteria for statistical significance was *P* < .006 (0.05/9 comparisons).

Abbreviations: C-MRI, conventional MRI; DL-MRI, deep learning MRI.

### Performance analysis of specific sequences

Analysis of sequences with relatively higher sampling (*n* ≥ 4) was performed (Table 4). The 2 highest-performing sequences were cervical axial T2 (*n* = 9) and brain axial T2 (*n* = 7). Cervical axial T2 achieved substantial time reduction (87.3 s, 67.3%) with high reader preference (3.81, 95% CI: 3.58–4.03), while brain axial T2 demonstrated the highest reader preference overall (3.89, 95% CI: 3.63–4.16) despite more modest time savings (46.0 s, 47.9%).

**Table 4.** Comparison of conventional MRI (C-MRI) and deep learning MRI (DL-MRI) acquisition time and image quality for sequences with N ≥ 4 pairs.

Sequence type and location	*N*	Average time reduction, seconds (%)	Signal-to-noise rating, mean (95% CI)	Overall preference, mean (95% CI)
Brain axial T2	7	46.0 (47.9%)	3.75 (3.50–4.00)	3.89 (3.63–4.16)
Cervical axial T2	9	87.3 (67.3%)	3.78 (3.58–4.03)	3.81 (3.58–4.03)
Brain sagittal T1	4	51.0 (53.8%)	3.69 (3.37–4.01)	3.62 (3.36–3.89)
Thoracic axial T2	6	48.0 (45.9%)	3.33 (3.08–3.51)	3.40 (2.99–3.81)
Brain axial T1	4	48.2 (49.6%)	3.50 (3.16–3.84)	3.56 (3.29–3.84)
Brain axial T2 FLAIR	4	27.0 (37.5%)	3.25 (2.75–3.75)	3.50 (3.23–3.90)
Cervical sagittal STIR	9	71.6 (55.6%)	3.44 (3.20–3.69)	3.47 (3.20–3.75)
Thoracic sagittal STIR	5	48.7 (41.1%)	3.12 (2.84–3.41)	3.12 (2.84–3.41)

For signal-to-noise rating and overall preference, 3 indicates equivalence between DL-MRI and C-MRI, numbers greater than 3 indicate preference for DL-MRI, and numbers less than 3 indicate preference for C-MRI.

Abbreviations: C-MRI, conventional MRI; DL-MRI, deep learning MRI.

The lowest performing sequence was thoracic sagittal STIR (*n* = 6), which showed moderate time reduction (48.7 s, 47.1%) but lower reader preference (3.12, 95% CI: 2.84–3.41). Wilcoxon rank sum testing demonstrated a significant difference between the highest-performing sequence (brain axial T2) and the lowest-performing sequence (thoracic sagittal STIR) (*P* = .002, effect size *r* = 0.47), suggesting that the benefits of DL-MRI could vary by sequence type and anatomical region (Figure S3).

T2-weighted sequences consistently outperformed other sequence types, particularly in axial orientation. While STIR sequences achieved substantial time reductions (48–72 s), they consistently received lower preference ratings (thoracic STIR: 3.12, 95% CI: 2.84–3.41; cervical STIR (*n* = 9): 3.47, 95% CI: 3.20–3.75).

### Interrater reliability

Interrater reliability was assessed using a single fixed raters model ICC (ICC3). The overall magnitude of reliability was low across all assessed parameters, with ICC values ranging from 0.064 to 0.329. The highest reliability was observed for overall preference (ICC = 0.329) and signal-to-noise rating (ICC = 0.293) (Table S4). Lower reliability was seen for tissue-specific assessments such as muscle (ICC = 0.064) and fat characterization (ICC = 0.066), indicating greater variability in how raters evaluated these specific features (Table S4). One of our raters reported that describing preferences between sequences on certain tissue-specific parameters was similar to “splitting hairs.”

### Additional observations concerning artifacts and visualization of specific structures

**Figure 4.**
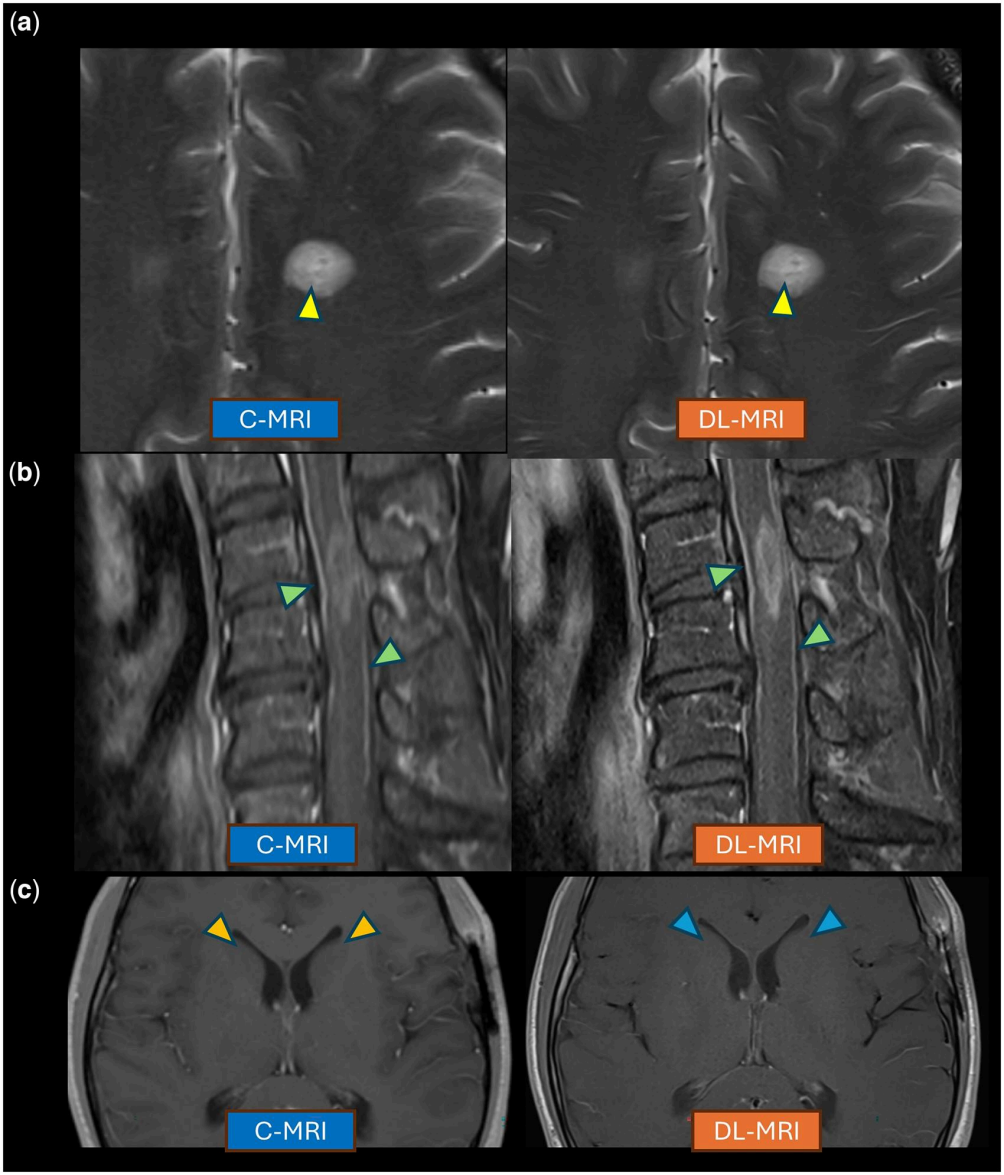
Observations on comparative visualization of specific structures on DL-MRI. (A) The central vein sign is more conspicuous on DL-MRI in an axial T2 image in a patient with multiple sclerosis (top row arrowheads). (B) The anterior and posterior median spinal veins are better defined on a sagittal post-contrast T1 image with DL-MRI (middle row arrowheads). (C) Differentiation of gray and white matter is less conspicuous on axial T1 weighted images of the brain on DL-MRI (bottom right arrowhead) compared to C-MRI (bottom left arrowhead). Abbreviations: C-MRI, conventional MRI; DL-MRI, deep learning MRI.

Through freeform text comments, raters also shared observations concerning artifacts and visualization of specific structures. The central vein sign was well visualized on axial T2 fat-saturated images in a patient with multiple sclerosis (Figure 4A), and the posterior median spinal vein was prominently seen on sagittal post-contrast T1 images (Figure 4B). Differentiation of gray and white matter was noted to be less conspicuous on axial T1-weighted images of the brain (Figure 4C).

**Figure 5.**
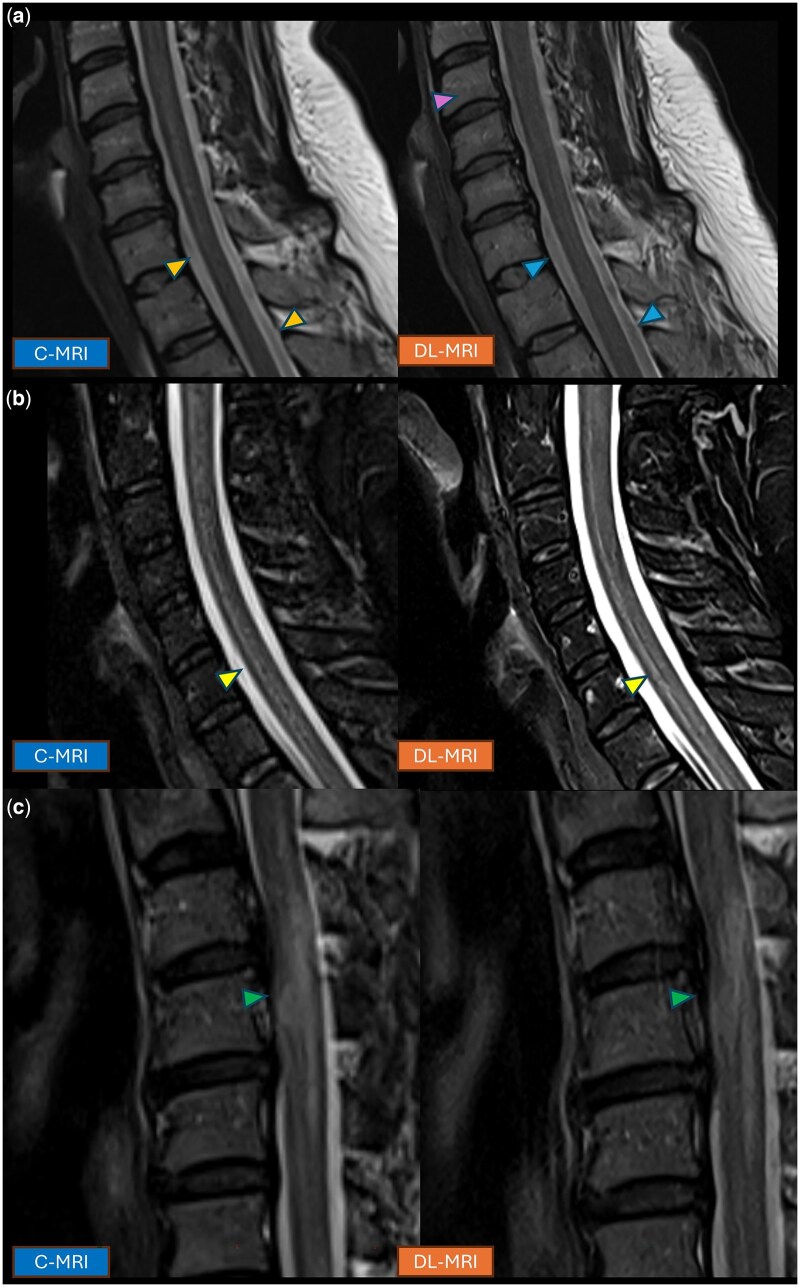
Paired sagittal T2-weighted images of the cervical spine in 3 separate patients acquired using C-MRI (left) and DL-MRI (right). (A) Cerebrospinal fluid-pulsation artifact was more conspicuous on DL-MRI (top right image, lower arrowheads) compared to C-MRI (top left image, lower arrowheads). Additionally, vertical banding artifacts in the phase-encoding direction were noted in the vertebral bodies on DL-MRI (top right image, top arrowhead). (B) Gibbs truncation artifact at the CSF-cord interface was more prominent on DL-MRI (middle row arrowheads). (C) On certain sequences the cord demonstrated a “grainier” texture on DL-MRI (lower row arrowheads). Abbreviations: C-MRI, conventional MRI; DL-MRI, deep learning MRI.

Raters also noted distinct artifact patterns with DL-MRI, particularly in spinal imaging. These included increased conspicuity of CSF pulsation artifact in sagittal T2-weighted sequences of the spine (Figure 5A), vertical banding artifacts in the phase-encoding direction on spinal imaging^25^ (Figure 5A), and more prominent Gibbs ringing artifact in the spinal cord (Figure 5B). Some raters noted that the spinal cord appeared “grainier” on certain DL-MRI sequences (Figure 5C). Although these were separate acquisitions, some of the DL-MRI sequences were also noted to exhibit slightly more motion artifact. Despite these artifacts, artifact ratings did not significantly differ between DL-MRI and C-MRI. The frequency of these artifacts was not specifically assessed.

## Discussion

Our head-to-head comparison of DL-MRI and C-MRI across a spectrum of neuroimaging examinations demonstrated that DL-MRI achieved an average 51.6% reduction in acquisition time through increased parallel acceleration and fewer averages. While average ratings modestly favored DL-MRI, interrater reliability was low, suggesting that clinically meaningful differences were not reliably apparent to the readers. These findings suggest that substantial time savings were achieved without compromising image quality.

### Scan time reduction

The substantial decrease in scan time offers significant potential advantages for patient care. Faster scans may expedite scheduling and reduce discomfort in patients with pain, altered mental status, or claustrophobia, while decreasing motion artifacts and repeat exams. Critically ill patients would spend less time in the scanner, reducing the risk of catastrophic events in a setting with limited options for rapid interventions. Shorter scan times may reduce or eliminate the need for anesthesia in certain patients, reducing logistical considerations and costs. Increased scanner throughput could also improve patient flow in busy inpatient/emergency departments, reducing wait time and length of stay.

### Overall image quality

While image quality assessments were slightly above neutral in favor of DL-MRI, the low interrater reliability suggested that there was no clinically meaningful difference between C-MRI and DL-MRI images across a spectrum of neuroradiologic sequences while providing substantial time savings. The time savings may be used to enable additional quality enhancement through protocol modifications. One example of a potential modification could involve reduction of slice thickness. Kim et al.^29^ showed that the use of 1-mm slice thickness MRI with DL-MRI significantly improved detection of cavernous sinus invasion compared to 3-mm slices. Imaging protocols could also use the time saved to add additional sequences or potentially employ more time-consuming sequences such as 3D sequences.

#### Sequence-specific findings

Time reduction varied substantially across sequence types. Although numerical differences in reader ratings were observed across individual sequences, the low interrater reliability and limited sample sizes limited meaningful interpretation of these variations. Further investigation with larger, more balanced cohorts would be needed to assess whether sequence-specific differences can inform the development of optimized hybrid protocols.

Our raters also provided qualitative observations. Gray–white differentiation on T1WI suffered with DL-MRI, while the central vein sign and posterior median spinal vein were better seen on DL-MRI. Comparative characterization of these specific structures could influence the application of DL-MRI in specific protocols where these structures would be of clinical interest. For example, our current DL-MRI sequences may be less optimal in cases where fine gray-white differentiation is of particular interest (such as in epilepsy protocols); however, they would be preferred for evaluating multiple sclerosis or potential spinal vascular pathologies. Additionally, DL-MRI could be emphasized for postcontrast T1WI where gray-white differentiation is less important.

#### Artifacts

While there was no significant difference in rater preference between DL-MRI and C-MRI with respect to artifacts (mean rating: 3.06, 95% CI: 3.00–3.13), we observed distinct artifact patterns that merit consideration in clinical practice. While the vertical banding represents a DL-MRI-specific artifact,^25^ increased conspicuity of conventional artifacts may be partially attributable to the enhanced sharpness inherent to DL-MRI, which can exaggerate structural edges of both tissue and artifacts. Although not explicitly reported in the free-text comments, this sharpness may have also contributed to more favorable structural delineation ratings for DL-MRI. While these artifacts did not significantly impact our raters’ assessment of diagnostic quality, awareness of these artifacts is important to prevent undue impact on interpretation.

Some authors have raised concerns that undersampling and reconstruction may result in loss or masking of pathology.^25^^,^^30^ While we did not observe clear instances of pathology being obscured in our cohort, this theoretical consideration has led some at our institution to favor a hybrid approach that combines both C-MRI and DL-MRI sequences. For instance, based on our exploratory findings, a hybrid approach implementing DL-MRI for T2-weighted sequences while maintaining conventional acquisition for STIR sequences could be reasonable at our institution, as T2-weighted sequences showed possibly high rater preference and substantial time savings with DL-MRI, while STIR sequences showed lower rater preference with more modest time reduction.

### Limitations and next steps

Our study’s single institution design utilizing a single 3 T Siemens scanner and vendor-specific DL software (Deep Resolve) with preliminarily optimized protocols limits generalizability. Multi-center validation studies with diverse vendors, scanner types, field strengths, and DL-MRI reconstruction algorithms would be useful to explore broader applicability. Our interrater reliability was low and better for global than tissue-specific assessments. This may partly reflect ratings clustering around the neutral value.^31^ This central tendency itself may be interpreted as evidence of functional equivalence between DL-MRI and C-MRI as raters often indicated no clear preference. Rater bias is another potential confounder, as distinct characteristics of DL-MRI, such as artifact patterns and enhanced image sharpness, together with readers’ varying levels of familiarity with DL-MRI, may have allowed raters to infer the reconstruction method. Another limitation is that our measures were not formally calibrated and assessed subjective preference rather than objective predefined criteria validated against established ground truth. Convenience sampling of diverse protocols allowed us to assess DL-MRI’s overall impact on routine clinical workflow, but limited detailed subgroup analyses. The heterogeneous nature of our data, with some protocols including only single patients (Supplementary Table S2), may have also contributed to the observed variance and low interobserver agreement. Additionally, it is possible that certain patients themselves could have been better suited to DL-MRI. Future studies would benefit from larger, more balanced datasets with more subjects per protocol to enable more robust analyses of relative performance between specific sequences. This would also help assess potential optimal hybrid protocols consisting of both C-MRI and DL-MRI sequences.

## Conclusions

Implementing deep learning in MRI reconstruction achieved a 51.6% reduction in acquisition time across a spectrum of neuroimaging studies at our institution without compromising image quality. These advancements have the potential to significantly enhance patient satisfaction, safety, and overall quality of care while offering substantial benefits to healthcare workflows and resource utilization.

Our overall findings support functional equivalence in imaging quality between C-MRI and DL-MRI in routine neuroradiologic imaging. While exploratory analyses raise the possibility that certain sequences may benefit disproportionately from DL-MRI, limited reliability and sequence-specific sample sizes preclude confident interpretation. Future studies with larger balanced cohorts are needed to determine whether there are optimal hybrid implementation strategies.

## Supplementary Material

umaf029_Supplementary_Data

## Data Availability

The data supporting this study are available from the corresponding author upon reasonable request. Patient-level data are anonymized and not publicly available due to privacy restrictions.
