# Supplementary material for: Deep learning MRI halves scan time and preserves image quality across routine neuroradiologic examinations
Source: Radiol Adv. 2025 Aug 23;2(5):umaf029. doi: 10.1093/radadv/umaf029 (PMC12483150; doi:10.1093/radadv/umaf029)

SUPPLEMENTARY MATERIAL

TITLE

Deep Learning MRI Halves Scan Time and Preserves Image Quality Across Routine Neuroradiologic Examinations

AUTHORS AND AFFILIATIONS

Shawn K. Lyo, MD[1]*

Suyash Mohan, MD[1]

Michael J. Hoch, MD[1+]

Vivek P. Patel, MD, PhD[1]

Robert M. Kurtz, MD[1]

Alvand Hassankhani, MD[1]

[1] Department of Radiology, Hospital of the University of Pennsylvania, Philadelphia, PA, USA

[+] Current affiliation: Department of Radiology, Thomas Jefferson University, Philadelphia, PA, USA

*Corresponding author

CORRESPONDING AUTHOR

Shawn Lyo, MD

Department of Radiology

Hospital of the University of Pennsylvania

3400 Spruce St., 1 Silverstein

Philadelphia, PA 19104, USA

Phone: +1 347 255 6737

Email: shawn.lyo@pennmedicine.upenn.edu

Table S1. Table of assessed parameters and instructions given to raters. Raters were asked to compare pairs of MRI sequences acquired using deep learning and conventional methods and indicate their preference for each parameter listed using the provided instructions.

**Parameter**	**Instructions**
Signal to Noise	Which sequence do you prefer for signal to noise ratio?
Structural Delineation	Which sequence better delineates anatomic structures?
Diagnostic Quality	Which sequence has higher diagnostic quality?
Presence of Artifacts	Which sequence is less degraded by artifacts?
Overall Preference	Which sequence do you prefer overall?
Fat Characterization	On which sequence is the fat better evaluated?
Muscle Characterization	On which sequence are the muscles better evaluated?
Osseous Characterization	On which sequence are the marrow signal and cortex better evaluated?
Neural Parenchymal Characterization	On which sequence are the neural structures better evaluated (i.e. brain, spinal cord parenchyma, etc.)?

Table S2. Table of C-MRI/DL-MRI sequence pairs by body part, orientation, and sequence type demonstrating number of each pair, acquisition time, scan time reduction, and raters' signal to noise rating and overall preference.

Abbreviations: C-MRI: Conventional MRI; DL-MRI: Deep Learning MRI; TMJ = Temporomandibular joint; IAC = Internal auditory canals; STIR = Short Tau Inversion

Recovery; FLAIR = Fluid-Attenuated Inversion Recovery; PD = Proton Density; Cor: Coronal; Ax: Axial; Sag: Sagittal;

**Body Part**	**Orientation**	**Sequence**	**N**	**Conventional Time, mean (SD)**	**DL Time, mean (SD)**	**Reduction, seconds (%)**	**Signal-to-Noise Rating, mean (95% CI)**	**Overall Preference, mean (95% CI)**	**Diagnostic Quality, mean (95% CI)**
Brachial Plexus			4	126.2 (80.3)	53.2 (33.0)	73.0s (57.8%)	3.50 (3.22 - 3.78)	3.62 (3.30-3.95)	3.31 (2.99-3.63)
	Ax		1	112.0 (nan)	31.0 (nan)	81.0s (72.3%)	3.50 (2.58 - 4.42)	3.50 (2.58-4.42)	3.50 (2.58-4.42)
		T1	1	112.0 (nan)	31.0 (nan)	81.0s (72.3%)	3.50 (2.58	3.50 (2.58-4.42)	3.50 (2.58-4.42)

							- 4.42)		
	Cor		1	232.0 (nan)	95.0 (nan)	137.0s (59.1%)	3.75 (2.95 - 4.55)	4.00 (2.70- 5.30)	3.50 (2.58- 4.42)
		T1	1	232.0 (nan)	95.0 (nan)	137.0s (59.1%)	3.75 (2.95 - 4.55)	4.00 (2.70- 5.30)	3.50 (2.58- 4.42)
	Sag		2	80.5 (61.5)	43.5 (29. 0)	37.0s (46.0%)	3.38 (2.94 - 3.81)	3.50 (3.05- 3.95)	3.12 (2.59- 3.66)
		STIR	1	124.0 (nan)	64.0 (nan)	60.0s (48.4%)	3.25 (2.45 - 4.05)	3.50 (2.58- 4.42)	3.25 (2.45- 4.05)
		T1	1	37.0 (nan)	23.0 (nan)	14.0s (37.8%)	3.50 (2.58 - 4.42)	3.50 (2.58- 4.42)	3.00 (1.70- 4.30)

Brain			28	93.4 (38.4)	50.8 (17.7)	42.6s (45.6%)	3.52 (3.38 - 3.66)	3.57 (3.43-3.71)	3.34 (3.24-3.44)
	Ax		18	85.3 (15.8)	48.1 (2.7)	37.2s (43.6%)	3.42 (3.23 - 3.60)	3.53 (3.34-3.71)	3.32 (3.20-3.44)
		T1	4	97.2 (13.7)	49.0 (0.0)	48.2s (49.6%)	3.50 (3.16 - 3.84)	3.56 (3.29-3.84)	3.44 (3.16-3.71)
		T1 POST	3	62.0 (0.0)	46.7 (4.0)	15.3s (24.7%)	2.75 (2.27 - 3.23)	2.58 (2.08-3.09)	3.00 (2.73-3.27)
		T2	7	96.0 (0.0)	50.0 (1.9)	46.0s (47.9%)	3.75 (3.50 - 4.00)	3.89 (3.63-4.16)	3.43 (3.21-3.65)
		T2 FLAIR	4	72.0 (0.0)	45.0 (0.0)	27.0s (37.5%)	3.25 (2.75	3.56 (3.23-3.90)	3.25 (3.01-3.49)

							- 3.75)		
	Cor		6	117.0 (78.3)	63.3 (37.4)	53.7s (45.9%)	3.71 (3.42 - 4.00)	3.67 (3.35-3.99)	3.38 (3.10-3.65)
		T1 POST	4	66.5 (3.7)	42.2 (2.5)	24.3s (36.5%)	3.75 (3.39 - 4.11)	3.88 (3.49-4.26)	3.44 (3.10-3.77)
		T2	2	218.0 (0.0)	105.5 (40.3)	112.5s (51.6%)	3.62 (3.00 - 4.25)	3.25 (2.66-3.84)	3.25 (2.66-3.84)
	Sag		4	94.8 (9.4)	43.8 (5.7)	51.0s (53.8%)	3.69 (3.37 - 4.01)	3.62 (3.36-3.89)	3.38 (3.11-3.64)
		T1	4	94.8 (9.4)	43.8 (5.7)	51.0s (53.8%)	3.69 (3.37 - 4.01)	3.62 (3.36-3.89)	3.38 (3.11-3.64)

Cervical spine			24	121.9 (21.0)	50.5 (10.8)	71.4s (58.6%)	3.55 (3.41-3.69)	3.55 (3.39-3.71)	3.27 (3.14-3.40)
	Ax		9	129.7 (17.3)	42.4 (5.9)	87.3s (67.3%)	3.78 (3.58-3.98)	3.81 (3.58-4.03)	3.39 (3.19-3.59)
		T2	9	129.7 (17.3)	42.4 (5.9)	87.3s (67.3%)	3.78 (3.58-3.98)	3.81 (3.58-4.03)	3.39 (3.19-3.59)
	Sag		15	117.2 (22.2)	55.4 (10.3)	61.8s (52.7%)	3.42 (3.23-3.60)	3.40 (3.19-3.61)	3.20 (3.04-3.36)
		STIR	9	128.8 (13.6)	57.2 (3.5)	71.6s (55.6%)	3.44 (3.20-3.69)	3.47 (3.20-3.75)	3.31 (3.07-3.55)
		T1	2	84.0 (0.0)	46.0 (0.0)	38.0s (45.2%)	3.25 (2.86	3.38 (2.75-4.00)	3.00 (2.55-3.45)

							- 3.64)		
		T1 POST	2	87.5 (2.1)	39.0 (4.2)	48.5s (55.4%)	3.88 (3.34 - 4.41)	3.50 (2.73-4.27)	3.12 (2.83-3.42)
		T2	2	128.0 (0.0)	73.0 (0.0)	55.0s (43.0%)	3.00 (2.37 - 3.63)	3.00 (2.37-3.63)	3.00 (2.55-3.45)
IAC			5	88.2 (21.0)	45.8 (4.9)	42.4s (48.1%)	3.15 (2.71 - 3.59)	3.15 (2.71-3.59)	3.05 (2.81-3.29)
	Ax		3	80.3 (11.2)	48.7 (3.2)	31.6s (39.4%)	3.08 (2.39 - 3.77)	3.17 (2.51-3.82)	3.08 (2.76-3.41)
		T1 POST	1	76.0 (nan)	50.0 (nan)	26.0s (34.2%)	3.00 (3.00 - 3.00)	3.00 (3.00-3.00)	3.00 (3.00-3.00)

		T2	1	93.0 (nan)	51.0 (nan)	42.0s (45.2%)	4.25 (2.73 - 5.77)	4.25 (2.73-5.77)	3.50 (2.58-4.42)
		T2 FLAIR	1	72.0 (nan)	45.0 (nan)	27.0s (37.5%)	2.00 (2.00 - 2.00)	2.25 (1.45-3.05)	2.75 (1.95-3.55)
	Cor		1	123.0 (nan)	44.0 (nan)	79.0s (64.2%)	2.75 (1.95 - 3.55)	2.50 (1.58-3.42)	2.75 (1.95-3.55)
		T1 POST	1	123.0 (nan)	44.0 (nan)	79.0s (64.2%)	2.75 (1.95 - 3.55)	2.50 (1.58-3.42)	2.75 (1.95-3.55)
	Sag		1	77.0 (nan)	39.0 (nan)	38.0s (49.4%)	3.75 (2.95 - 4.55)	3.75 (2.95-4.55)	3.25 (2.45-4.05)
		T1 POST	1	77.0 (nan)	39.0 (nan)	38.0s (49.4%)	3.75 (2.95	3.75 (2.95-4.55)	3.25 (2.45-4.05)

							- 4.55)		
Lumbar spine			14	101.2 (21.8)	54.6 (14.1)	46.6s (46.0%)	3.38 (3.19 - 3.56)	3.48 (3.32-3.64)	3.29 (3.16-3.41)
	Ax		5	86.4 (28.4)	47.2 (16.2)	39.2s (45.4%)	3.30 (2.99 - 3.61)	3.45 (3.21-3.69)	3.25 (3.04-3.46)
		T1	2	55.5 (3.5)	29.5 (2.1)	26.0s (46.8%)	3.62 (3.19 - 4.06)	3.50 (3.05-3.95)	3.25 (2.86-3.64)
		T2	3	107.0 (3.5)	59.0 (1.7)	48.0s (44.9%)	3.08 (2.66 - 3.51)	3.42 (3.09-3.74)	3.25 (2.96-3.54)
	Sag		9	109.4 (12.4)	58.8 (11.8)	50.6s (46.3%)	3.42 (3.18 - 3.65)	3.50 (3.28-3.72)	3.31 (3.15-3.46)

		STIR	3	119.0<br>(12.1)	51.3<br>(6.0)	67.7s<br>(56.9%)	3.17<br>(2.71<br>-<br>3.62)	3.17<br>(2.71-<br>3.62)	3.17<br>(2.92-<br>3.41)
		T1	3	108.0<br>(10.4)	70.7<br>(13.<br>3)	37.3s<br>(34.5%)	3.67<br>(3.25<br>-<br>4.08)	3.75<br>(3.36-<br>4.14)	3.50<br>(3.17-<br>3.83)
		T2	3	101.3<br>(11.0)	54.3<br>(4.0)	47.0s<br>(46.4%)	3.42<br>(2.99<br>-<br>3.84)	3.58<br>(3.26-<br>3.91)	3.25<br>(2.96-<br>3.54)
Neck			5	76.6 (14.9)	47.6<br>(5.8)	29.0s<br>(37.9%)	3.60<br>(3.25<br>-<br>3.95)	3.70<br>(3.30-<br>4.10)	3.45<br>(3.13-<br>3.77)
	Ax		3	77.3 (9.9)	47.0<br>(7.9)	30.3s<br>(39.2%)	3.33<br>(2.84<br>-<br>3.83)	3.58<br>(3.08-<br>4.09)	3.50<br>(3.07-<br>3.93)
		STIR	1	66.0 (nan)	53.0<br>(nan<br>)	13.0s<br>(19.7%)	3.75<br>(2.95	3.75<br>(2.95-<br>4.55)	3.50<br>(2.58-<br>4.42)

							- 4.55)		
		T1	1	84.0 (nan)	50.0 (nan)	34.0s (40.5%)	3.50 (2.58 - 4.42)	3.75 (2.23-5.27)	3.75 (2.23-5.27)
		T2	1	82.0 (nan)	38.0 (nan)	44.0s (53.7%)	2.75 (1.23 - 4.27)	3.25 (1.73-4.77)	3.25 (2.45-4.05)
	Cor		1	57.0 (nan)	47.0 (nan)	10.0s (17.5%)	3.75 (2.95 - 4.55)	3.50 (1.45-5.55)	3.50 (1.91-5.09)
		T1 POST	1	57.0 (nan)	47.0 (nan)	10.0s (17.5%)	3.75 (2.95 - 4.55)	3.50 (1.45-5.55)	3.50 (1.91-5.09)
	Sag		1	94.0 (nan)	50.0 (nan)	44.0s (46.8%)	4.25 (3.45 - 5.05)	4.25 (3.45-5.05)	3.25 (2.45-4.05)

		T1	1	94.0 (nan)	50.0 (nan)	44.0s (46.8%)	4.25 (3.45 - 5.05)	4.25 (3.45-5.05)	3.25 (2.45-4.05)
Orbits			6	136.0 (53.7)	63.0 (27.5)	73.0s (53.7%)	3.71 (3.39 - 4.03)	3.92 (3.64-4.19)	3.58 (3.31-3.86)
	Ax		2	86.0 (9.9)	86.5 (17.7)	-0.5s (-0.6%)	3.50 (2.87 - 4.13)	3.75 (3.36-4.14)	3.38 (2.94-3.81)
		T1	1	93.0 (nan)	74.0 (nan)	19.0s (20.4%)	3.75 (2.95 - 4.55)	3.50 (2.58-4.42)	3.25 (2.45-4.05)
		T1 POST	1	79.0 (nan)	99.0 (nan)	-20.0s (-25.3%)	3.25 (1.73 - 4.77)	4.00 (4.00-4.00)	3.50 (2.58-4.42)
	Cor		4	161.0 (47.6)	51.2 (24.6)	109.8s (68.2%)	3.81 (3.41	4.00 (3.61-4.39)	3.69 (3.31-4.06)

							- 4.21)		
		STIR	1	196.0 (nan)	35.0 (nan)	161.0s (82.1%)	3.50 (2.58 - 4.42)	3.50 (2.58-4.42)	3.50 (2.58-4.42)
		T1	1	121.0 (nan)	87.0 (nan)	34.0s (28.1%)	4.00 (2.70 - 5.30)	4.25 (3.45-5.05)	3.25 (2.45-4.05)
		T1 POST	1	119.0 (nan)	48.0 (nan)	71.0s (59.7%)	4.25 (2.73 - 5.77)	4.75 (3.95-5.55)	4.50 (3.58-5.42)
		T2	1	208.0 (nan)	35.0 (nan)	173.0s (83.2%)	3.50 (2.58 - 4.42)	3.50 (2.58-4.42)	3.50 (2.58-4.42)
Sella			5	106.6 (28.1)	28.2 (7.4)	78.4s (73.5%)	4.15 (3.84 - 4.46)	4.05 (3.77-4.33)	3.55 (3.27-3.83)

	Cor		3	98.7 (4.0)	28.0 (10.4)	70.7s (71.6%)	4.17 (3.71-4.62)	4.25 (3.86-4.64)	3.67 (3.25-4.08)
		T1	1	101.0 (nan)	22.0 (nan)	79.0s (78.2%)	4.50 (3.58-5.42)	4.50 (3.58-5.42)	4.25 (3.45-5.05)
		T1 POST	1	101.0 (nan)	22.0 (nan)	79.0s (78.2%)	4.50 (3.58-5.42)	4.50 (3.58-5.42)	3.75 (2.95-4.55)
		T2	1	94.0 (nan)	40.0 (nan)	54.0s (57.4%)	3.50 (2.58-4.42)	3.75 (2.95-4.55)	3.00 (3.00-3.00)
	Sag		2	118.5 (51.6)	28.5 (2.1)	90.0s (75.9%)	4.12 (3.59-4.66)	3.75 (3.36-4.14)	3.38 (2.94-3.81)
		T1	1	82.0 (nan)	27.0 (nan)	55.0s (67.1%)	4.00 (2.70	3.50 (2.58-4.42)	3.25 (2.45-4.05)

							- 5.30)		
		T1 POST	1	155.0 (nan)	30.0 (nan)	125.0s (80.6%)	4.25 (3.45 - 5.05)	4.00 (4.00-4.00)	3.50 (2.58-4.42)
TMJ			6	179.7 (62.2)	83.2 (11.6)	96.5s (53.7%)	3.54 (3.24 - 3.85)	3.62 (3.30-3.95)	3.46 (3.21-3.71)
	Ax		1	64.0 (nan)	89.0 (nan)	-25.0s (-39.1%)	2.75 (1.23 - 4.27)	3.00 (1.16-4.84)	3.50 (2.58-4.42)
		T1	1	64.0 (nan)	89.0 (nan)	-25.0s (-39.1%)	2.75 (1.23 - 4.27)	3.00 (1.16-4.84)	3.50 (2.58-4.42)
	Cor		2	199.0 (0.0)	79.0 (0.0)	120.0s (60.3%)	3.50 (2.87 - 4.13)	3.62 (3.19-4.06)	3.50 (3.05-3.95)

		T1	2	199.0 (0.0)	79.0 (0.0)	120.0s (60.3%)	3.50 (2.87 - 4.13)	3.62 (3.19-4.06)	3.50 (3.05-3.95)
	Sag		3	205.3 (40.4)	84.0 (17.3)	121.3s (59.1%)	3.83 (3.59 - 4.08)	3.83 (3.38-4.29)	3.42 (2.99-3.84)
		PD	2	182.0 (0.0)	94.0 (0.0)	88.0s (48.4%)	3.88 (3.58 - 4.17)	3.88 (3.18-4.57)	3.50 (2.87-4.13)
		T2	1	252.0 (nan)	64.0 (nan)	188.0s (74.6%)	3.75 (2.95 - 4.55)	3.75 (2.95-4.55)	3.25 (2.45-4.05)
Thoracic spine			16	113.1 (32.8)	59.9 (10.7)	53.2s (47.0%)	3.38 (3.19 - 3.56)	3.34 (3.15-3.54)	3.28 (3.12-3.44)
	Ax		6	104.5 (28.8)	56.5 (7.4)	48.0s (45.9%)	3.55 (3.16	3.40 (2.99-3.81)	3.35 (3.00-3.70)

							- 3.94)		
		T2	6	104.5 (28.8)	56.5 (7.4)	48.0s (45.9%)	3.55 (3.16 - 3.94)	3.40 (2.99- 3.81)	3.35 (3.00- 3.70)
	Sag		1 1	117.7 (35.2)	61.8 (12. 0)	55.9s (47.5%)	3.30 (3.08 - 3.51)	3.32 (3.09- 3.54)	3.25 (3.06- 3.44)
		STIR	5	118.5 (46.7)	69.8 (5.3)	48.7s (41.1%)	3.12 (2.84 - 3.41)	3.12 (2.84- 3.41)	3.12 (2.87- 3.38)
		T1	2	128.5 (7.8)	60.0 (9.9)	68.5s (53.3%)	3.62 (3.19 - 4.06)	3.75 (3.36- 4.14)	3.50 (2.87- 4.13)
		T1 POST	1	90.0 (nan)	38.0 (nan)	52.0s (57.8%)	3.50 (2.58 - 4.42)	3.75 (2.95- 4.55)	3.25 (2.45- 4.05)

		T2	2	118.5 (19.1)	51.5 (6.4)	67.0s (56.5%)	3.38 (2.61 - 4.14)	3.25 (2.38-4.12)	3.38 (2.94-3.81)

Table S3. Comparison of scan parameters between DL-MRI and C-MRI. DL-MRI sequences were acquired 51.6% faster than C-MRI on average due to increased acceleration factor and fewer averages. (Following Bonferroni correction, adjusted statistical criteria for significance was p<0.005 (*)).

Abbreviations: : C-MRI: Conventional MRI; DL-MRI: Deep Learning MRI;

**Parameter**	**Conventional MRI (mean, SD)**	**Deep Learning MRI (mean, SD)**	**t-value**	**p-value**
TR (ms)	3327 (2485)	3170.42 (2323)	2.12	0.03
TE (ms)	50.81 (41.81)	50.80 (40)	0.01	0.99
Number of Averages*	1.88 (0.53)	1.11 (0.34)	13.84	<0.001*
Number of Phase Encoding Steps	246.60 (40.17)	245.02 (38.60)	0.64	0.52
Echo Train Length	10.53 (6.86)	10.76 (6.67)	-0.84	0.39
Percent Sampling	79.17 (7.70)	80.22 (7.54)	-2.31	0.02
SAR	0.90 (0.49)	0.94 (0.52)	-1.08	0.28

Parameter	Conventional MRI (mean, SD)	Deep Learning MRI (mean, SD)	t-value	p-value
Pixel Bandwidth (Hz/Px)	262.62 (52.88)	268.97 (53.23)	-2.72	0.007
Total Acquisition Time (s)*	110.84 (40.64)	53.65 (17.45)	16.82	<0.001*
Acceleration Factor*	1.54 (0.90)	3.58 (0.65)	-20.42	<0.001*

TE - Echo time; TR - Repetition time; Hz/Px – Hertz/Pixel; SAR: Specific absorption rate

Table S4. Analysis of interrater reliability.

Category	ICC3
Signal-to-Noise Rating	0.293
Diagnostic Quality Rating	0.115
Artifacts Rating	0.072
Structural Delineation Rating	0.133
Overall Preference Rating	0.329
Fat Rating	0.066
Muscle Rating	0.064
Marrow Rating	0.139

Category	ICC3
Neural Rating	0.166

Fig S1. Schematic demonstrating rating interface. Paired, fully interactive DICOM series were randomized and displayed side by side. Whether the C-MRI or DL-MRI sequences were displayed on the left or right was randomized. Blinded raters indicated a preference for either the series on the left or right using a Likert scale.

Abbreviations: C-MRI: Conventional MRI; DL-MRI: Deep Learning MRI

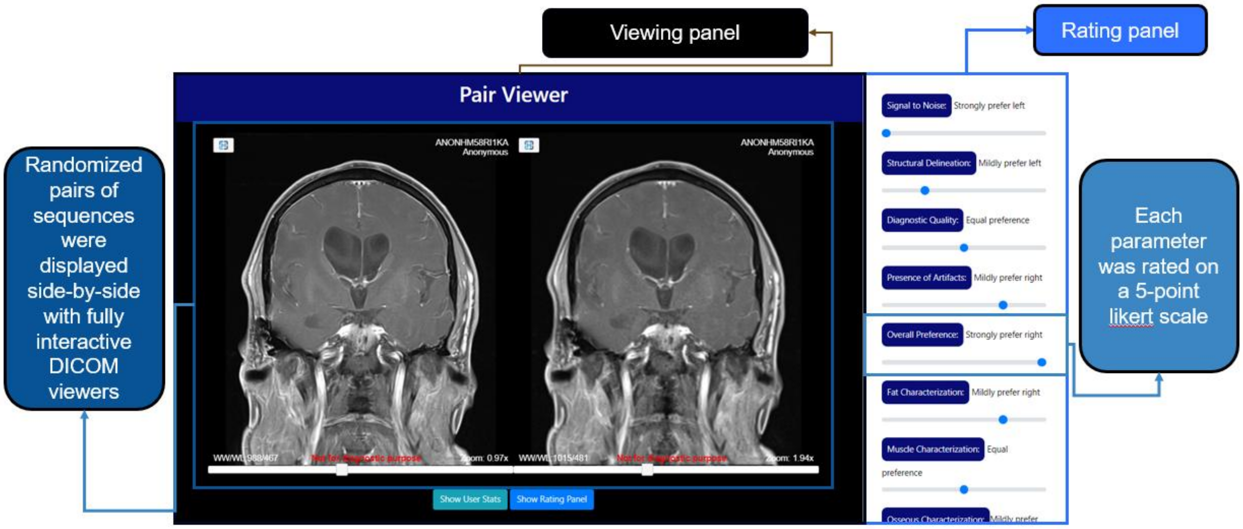

Fig S2. Comparison of mean scan time by various parameters, including body part (top), sequence orientation (middle), and sequence type (bottom). DL-MRI required an average of half the scan time of C-MRI, and the largest decrease in acquisition time involved sequences evaluating the sella and those acquired in the coronal plane. Error bars represent standard deviations.

Abbreviations: C-MRI: Conventional MRI; DL-MRI: Deep Learning MRI; TMJ = Temporomandibular joint; IAC = Internal auditory canals; STIR = Short Tau Inversion Recovery; FLAIR = Fluid-Attenuated Inversion Recovery; PD = Proton Density; Cor: Coronal; Ax: Axial; Sag: Sagittal

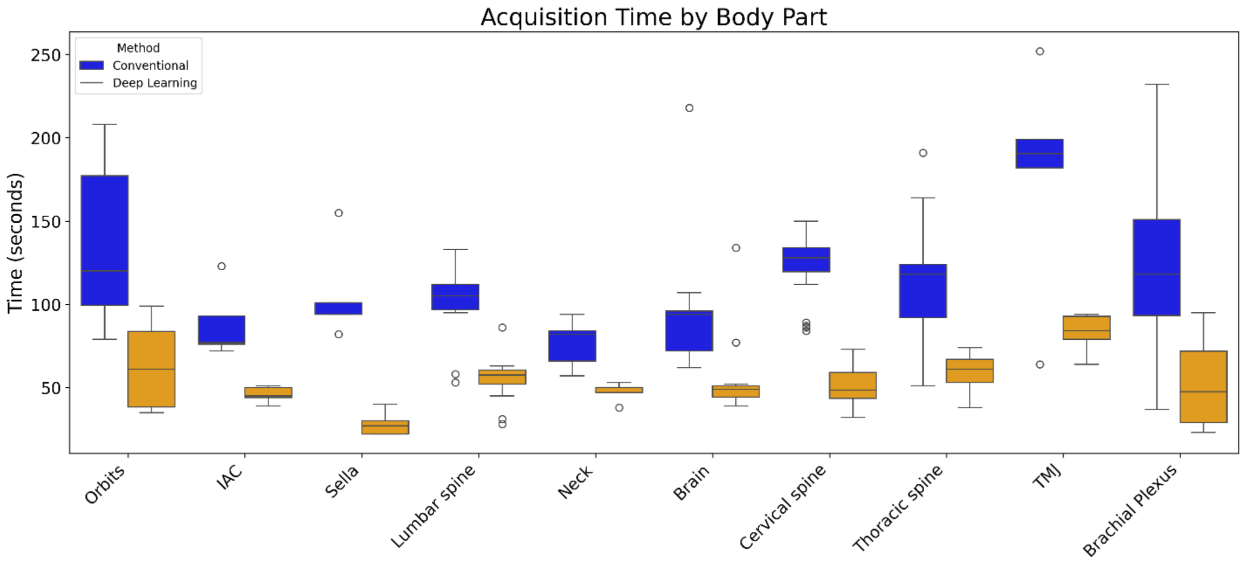
Acquisition Time by Body Part
Method
Conventional
Deep Learning
Time (seconds)
250
200
150
100
50
Orbits
IAC
Sella
Lumbar spine
Neck
Brain
Cervical spine
Thoracic spine
TMJ
Brachial Plexus

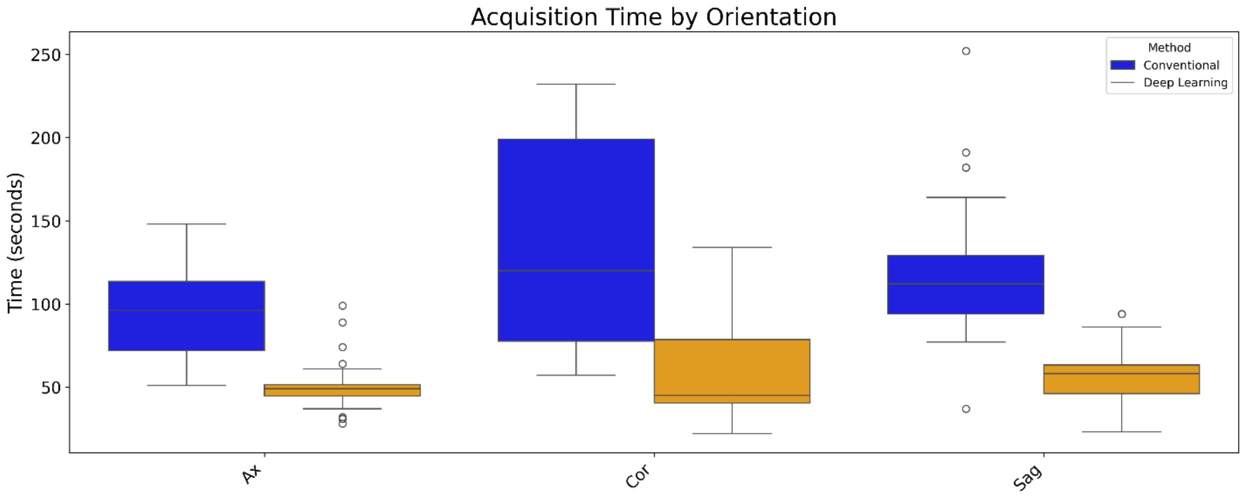
Acquisition Time by Orientation
Method
Conventional
Deep Learning
Time (seconds)
250
200
150
100
50
Ax
Cor
Sag

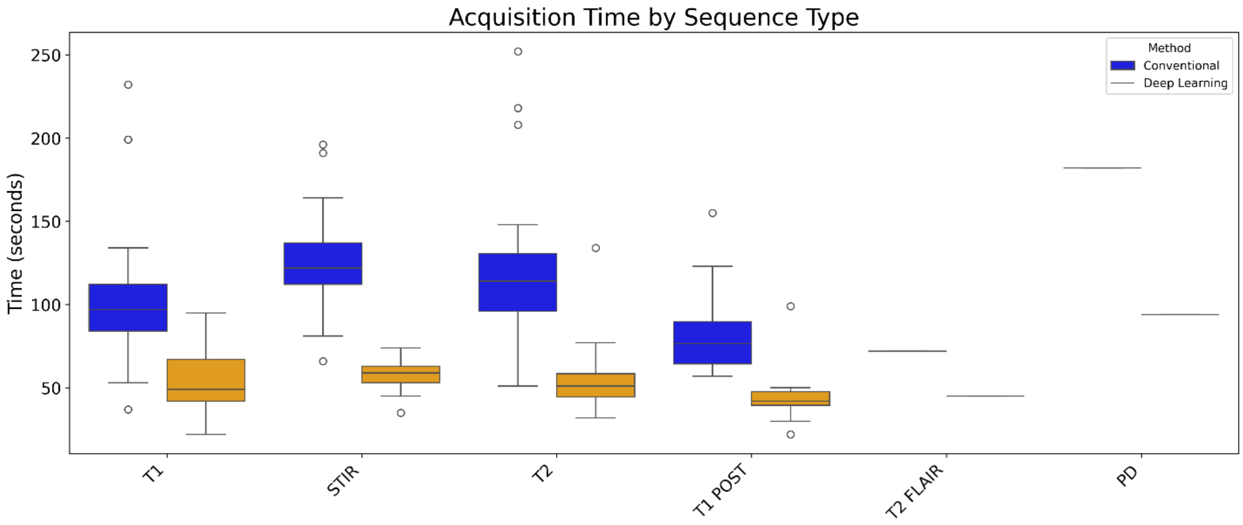
Acquisition Time by Sequence Type
Method
Conventional
Deep Learning
Time (seconds)
250
200
150
100
50
T1
STIR
T2
T1 POST
T2 FLAIR
PD

Fig S3. DL-MRI sequence performance map comparing time reduction and mean overall preference rating for sequences with N⩾4 pairs. Bubble size indicates number of sequence pairs. Dashed line at 3.0 represents neutral preference. Higher y-axis values indicate preference for DL-MRI over C-MRI and higher x-axis values indicate increased time reduction in seconds.

Abbreviations: C-MRI: Conventional MRI; DL-MRI: Deep Learning MRI;

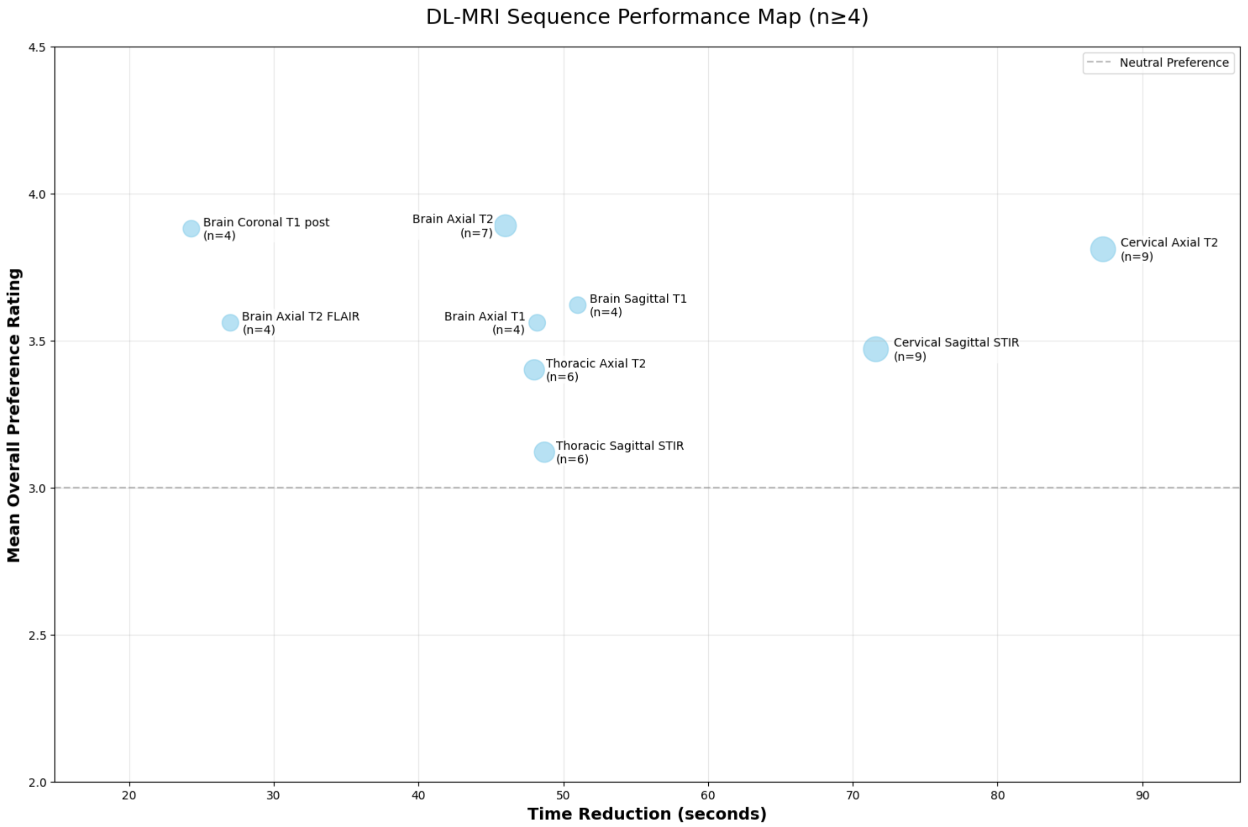

Supplement: umaf029_Supplementary_Data [file umaf029_supplementary_data.zip › Supplementary_Data.zip!/DeepLearningMRI-RA-Supplemental-Final.pdf]
